# Audit of the quality and content of discharge summaries from mental health inpatient units across Betsi Cadwaladr University Health Board

**DOI:** 10.1192/bjo.2021.241

**Published:** 2021-06-18

**Authors:** Asha Dhandapani, Sathyan Soundararajan, Laura Williams, Ediriverere Endurance Aghahowa, John Clifford

**Affiliations:** BCUHB

## Abstract

**Aims:**

Our aim was to carry out an audit of summaries sent from inpatient psychiatric units across North Wales (namely Heddfan in Wrexham, Ablett in Rhyl, and Hergest in Bangor), against recommendations from ‘Standards for Inpatient Mental Health Services’ (RCPsych 2014) and PRSB Mental Health Discharge guidelines (2018).

**Method:**

Ablett summaries are typed onto and electronically sent through the Welsh Clinical Portal (WCP) directly to the GP. Hergest and Heddfan both have their own templates which are then sent to the GP and filed in the case notes. Data were collected from both sources. The first audit cycle used 25 discharges selected at random from the male and female open wards in each site (n = 75 summaries). Data were collected over 3 months time using the audit proforma.

**Result:**

All mandatory headings are automatically inputted into the WCP summary used in Ablett therefore documentation was 100% for information such as patient name, DOB, and GP Details. Documentation of allergies was poor across 3 sites, particularly in Hergest, in which there was no mention of allergy status in 96% of summaries. Only 13% of Ablett summaries and 0% of Hergest summaries reach the GP on the day of discharge, however, 100% of summaries from Heddfan do, possibly due to their method of ‘discharge notification’. The date and location of discharge were documented in 84% of Heddfan summaries, 100% of Hergest summaries, and 100% of Ablett summaries. This implies that this heading is already incorporated into the templates for the 2 sites which scored 100%. In the Ablett, medication was documented in 88%, but we found that in 49% of discharge summaries, the medication was the only field filled in! In these cases, the GP may not even know why the patient had been admitted. This is clearly unacceptable. Risk history is poorly documented across the sites, with 0% in Hergest and Heddfan, and 12% in Ablett. 0% of summaries across the Health Board mentioned crisis contacts. 0% of summaries in Heddfan and Ablett contained details of the patient's care coordinator.

**Conclusion:**

Our audit has identified a lack of psychiatry-relevant headings in the discharge summaries, particularly for those working in Ablett.

